# Breast cancer: comparison of quantitative dual-layer spectral CT and axillary ultrasonography for preoperative diagnosis of metastatic axillary lymph nodes

**DOI:** 10.1186/s41747-021-00212-6

**Published:** 2021-04-09

**Authors:** Thomas Winther Buus, Mads Sandahl, Kennet Sønderstgaard Thorup, Finn Rasmussen, Søren Redsted, Peer Christiansen, Anders Bonde Jensen, Erik Morre Pedersen

**Affiliations:** 1 Department of Radiology, Aarhus University Hospital, Palle Juul-Jensens Boulevard 99, 8200 Aarhus N, Denmark; 2 Department of Plastic and Breast Surgery, Aarhus University Hospital, Palle Juul-Jensens Boulevard 35, 8200 Aarhus N, Denmark; 3 Department of Oncology, Aarhus University Hospital, Palle Juul-Jensens Boulevard 99, 8200 Aarhus N, Denmark

**Keywords:** Axilla, Breast neoplasms, Lymphatic metastasis, Tomography (x-ray, computed), Ultrasonography

## Abstract

**Background:**

Our aim was to compare the diagnostic performance of quantitative dual-layer spectral computed tomography (DLSCT) and axillary ultrasound (US) for diagnosing lymph node metastases in breast cancer patients.

**Methods:**

DLSCT and axillary US were prospectively performed in 70 needle biopsy-verified breast cancer patients. Histopathology and imaging data were available for evaluation in 36 axillae from 34 patients. In each patient, ipsilateral, contralateral, and inguinal lymph nodes (LNs) were semiautomatically segmented, and iodine density, spectral slope, *Z* effective, virtual non-contrast (VNC), conventional CT HU values, and Δ contrast enhancement (ΔCE, conventional CT HU minus VNC) were measured. Using histopathology as reference, the diagnostic performance of DLSCT and axillary US was compared.

**Results:**

Of 36 axillae, 23 had metastatic lymph nodes. Compared with non-metastatic LNs, metastatic LNs had significantly different iodine density (*p* = 0.021), spectral slope (*p* < 0.001), *Z* effective (*p* < 0.001), conventional CT HU values (*p* < 0.01), and ΔCE (*p* < 0.01). All DLSCT parameters were significantly different between arterial phase and portal-venous phase (*p* < 0.001) except for VNC (*p* = 0.092). ΔCE had the highest diagnostic performance (sensitivity 0.79, specificity 0.92, positive predictive value 0.95, negative predictive value 0.69) with a significantly increased sensitivity compared with conventional CT HU (*p* = 0.027). There were no significant differences between ΔCE and axillary US for sensitivity (*p* = 1.000) or specificity (*p* = 0.320).

**Conclusions:**

DLSCT is a promising quantitative technique for evaluating LN metastases and could potentially reduce the need for sentinel LN biopsy.

## Key points

- Quantitative parameters from dual-layer detector spectral computed tomography (DLSCT) can discriminate between metastatic and non-metastatic axillary lymph nodes.
- Amongst DLSCT parameters, the difference in HU between contrast-enhanced images and virtual non-contrast images (Δ contrast enhancement) had the highest diagnostic performance
- Quantitative parameters from DLSCT showed not significantly different sensitivity and specificity for diagnosing axillary lymph node metastases as ultrasonography with fine needle aspiration.

## Background

Breast cancer is the most common cancer affecting women and the second leading cause of cancer-related deaths [1]. The axillary lymph nodes are the most common site for metastatic disease [2]. Correct identification of axillary lymph node metastases is important for both determining the patient’s prognosis and for planning the patient’s treatment [3], as the presence of axillary lymph node metastases has a negative impact on overall survival [4].

Ultrasonography (US) of the breasts and axillae with fine needle aspiration (FNA) from morphologically suspicious axillary lymph nodes is often used for preoperative staging of the axilla. Sensitivity for detecting axillary lymph node metastases has been reported to be around 80% with specificity near 100% [5, 6]. US is usually followed by sentinel lymph node biopsy (SNB), which, if negative, rules out axillary lymph node metastases with high accuracy [7]. Patients with < 3 positive nodes on SNB can often be managed with SNB as the only surgical procedure in the axilla [8, 9]. However, patients with clinically positive lymph nodes, ≥ T3 tumour, ≥ 3 positive nodes on SNB, or planned mastectomy will often receive axillary lymph node dissection (ALND) [10]. Since both SNB and ALND are invasive surgical procedures (which may cause immediate complications such as infection or, in a few cases, long-term effects such as lymphedema, shoulder impairment, or pain [11–13]), non-invasive methods to reduce the need for SNB and ALND are highly desired.

Dual-energy computed tomography (DECT) is an emerging technique [14]. Where conventional CT describes the tissues relative x-ray attenuation properties measured in HU, the dual-energy approach provides quantitative measures of material concentrations and decompositions (*e.g*., iodine and uric acid) as well as the ability to suppress materials such as iodine, water, or calcium [15, 16].

Most dual-energy techniques acquire a spectral CT dataset by scanning the same area twice with different kilovolt peak. This can be achieved either by dual-source CT or by rapid tube potential switching (single-source) [14, 16, 17]. The improvement in iodine visualisation and quantification offered by DECT has shown promising results in the diagnosis of lymph node metastases from a range of different cancer diseases [18–22], including breast cancer [23]. However, this approach previously required that patients were selected prospectively for DECT and that a special DECT protocol was selected prior to scanning, limiting its clinical usefulness.

The newest generation of DECT systems uses a dual-layer detector [24] for separating the x-ray spectrum, with the top row detecting low-energy photons and the bottom layer detecting high-energy photons [14, 16, 17]. This removes the need for changing the tube kilovolt peak or other scan parameters whilst still having all spectral CT parameters available for analyses using a spectral workstation. In addition, the conventional CT images have been shown to not differ from single-energy CT in dose [25] or image quality [26]

The aim of the present study was to assess the diagnostic performance of quantitative spectral CT parameters from dual-layer (detector) spectral CT (DLSCT) to preoperatively diagnose axillary lymph node metastases in breast cancer patients undergoing definitive surgery. Quantitative spectral parameters were compared to axillary US using the results from the histopathological examination of the surgical specimen as reference.

## Methods

### Ethical approval and patient enrolment

This prospective study was approved by the Central Denmark Region Committee on Health Research Ethics (reference number 1-10-72-425-17). Written informed consent was obtained prior to the patients’ DLSCT examination.

The inclusion criteria were: women aged ≥ 18 years with needle biopsy-verified primary or recurrent breast cancer referred for diagnostic workup due to the potential risk of disseminated disease. Indications for referral for diagnostic workup included: new breast cancer with previous contralateral breast cancer, large breast tumour (≥ T3), carcinomatous mastitis, recurrent ipsilateral breast cancer, and suspicion of substantial axillary involvement. Eligible patients were identified at the Department of Plastic and Breast Surgery, Aarhus University Hospital, Denmark.

### Axillary ultrasonography

Axillary US was performed by subspecialised radiologists in a tertiary centre. The US examination was accomplished using a HI VISION Ascendus scanner with an 18-MHz linear transducer (Hitachi Medical Systems, Tokyo, Japan). The examination was performed according to the European Society of Breast Imaging guidelines [27]. Before US, inspection and palpation of both breast and axillae were performed. Afterwards, the patients had both breasts and axillae examined with US, and if any suspicious lymph nodes were detected, US-guided FNA was obtained in the most suspicious lymph node. The following criteria were used to determine whether a lymph node was considered suspicious [28]: cortex thickening > 3 mm (symmetric or asymmetric), peripheral vascularisation and oedema, perinodal growth, or hilar infiltration. In addition, morphologically benign-appearing lymph nodes, which were dissimilar to the contralateral ones (*e.g*., asymmetrical, puckered outline, or slight cortex thickening), were also selected for biopsy. Cytology of US-guided FNA and histopathology from SNB or ALND were performed per our pathology department’s standard.

### DLSCT protocol

Contrast-enhanced DLSCT of the chest, abdomen, and pelvis was acquired using a 64-row scanner (IQon, Philips Healthcare, Best, The Netherlands). The following acquisition parameters were used: 120–140 kVp, 150–250 mAs, rotation time 0.75 s, pitch 1.078, collimation 64 × 0.625 mm, matrix 512 × 512, reconstructed slice thickness 2 mm, and increment 1 mm. Iodixanol 320 mg/mL (Visipaque 320, General Electric Healthcare, Chicago, IL) was administered intravenously in the contralateral arm (28 patients), in the ipsilateral arm (5 patients), or in the intravenous port (1 patient) in weight-adjusted doses of 2 mL/kg with an injection rate of 4 mL/s. If the patient had previous adverse effect due to iodixanol, iohexol 350 mg/mL (Omnipaque 350, General Electric Healthcare, Chicago, IL) was used instead. A region of interest placed in the descending aorta was used for bolus tracking with a threshold of 150 HU. Chest and upper abdomen DLSCT scans were acquired in the arterial phase with a 15-s delay after reaching the threshold in the aorta. DLSCT scans of the abdomen and pelvis were acquired in the portal-venous phase with 65-s delay after reaching the threshold in the aorta. A DLSCT dataset was reconstructed by spectral separation of the two detector layers’ signals. In addition, a conventional CT dataset was reconstructed from weighted addition of the two detector layers’ signals. The average estimated effective radiation dose of the DLSCT examination was 21.5 mSv (range 17.5–27.6 mSv), in particular, 8 mSv (range: 6.3–11.9 mSv) for chest and upper abdomen and 13.5 mSv (11.3–16.5 mSv) for abdomen and pelvis.

### Image analysis

**Fig. 1:**
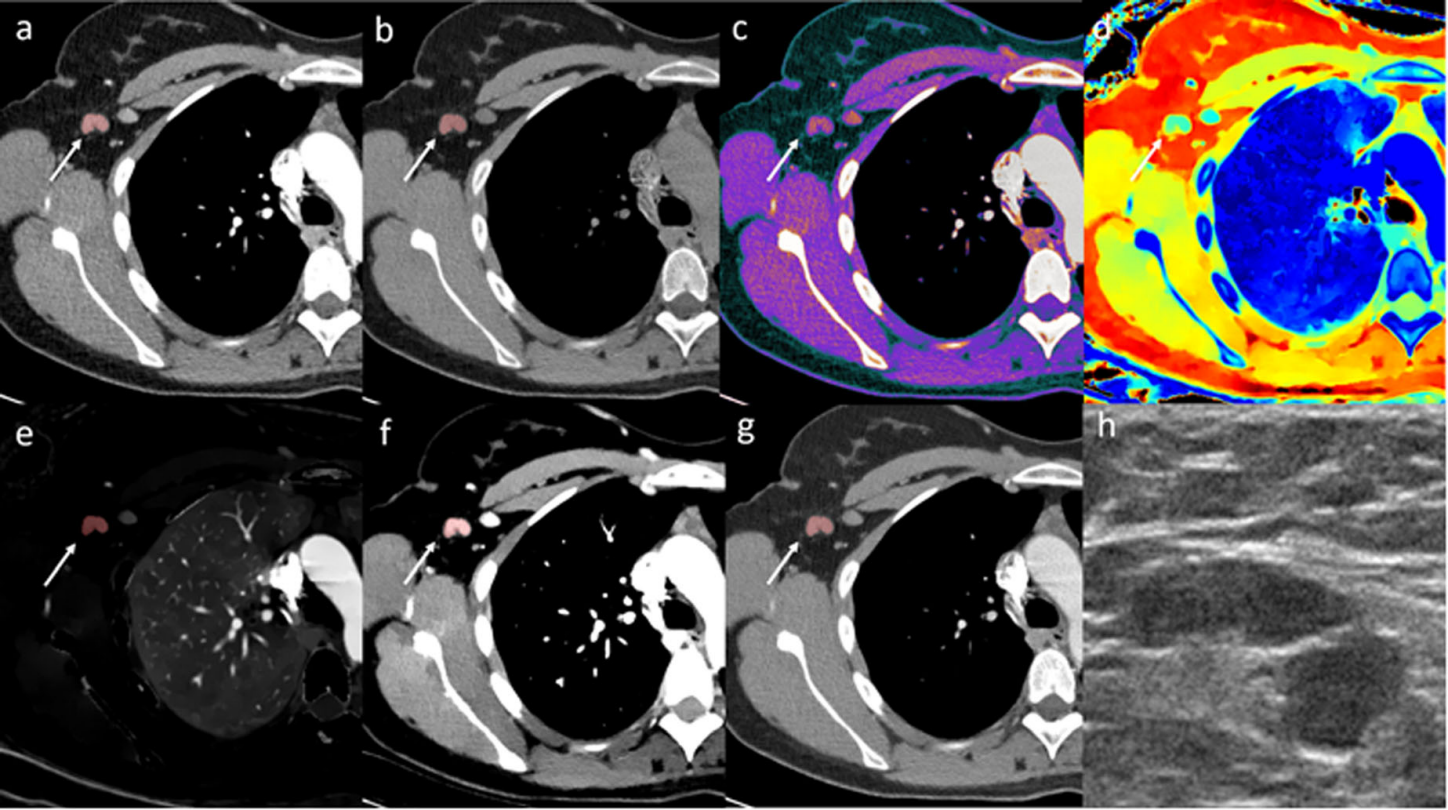
Arterial phase dual-layer detector spectral CT (DLSCT) examination of a woman with a suspicious lymph node in the right axilla. The lymph node shows high conventional CT HU values (**a**), Δ contrast enhancement (**c**) (conventional CT (HU) (**a**) − virtual non-contrast (HU) (**b**)), low Z effective (**d**), high iodine density (**e**), and spectral slope ((mono-energy 40 keV (**f**) minus mono-energy 100 keV (**g**))/60 keV). Based on the cutoff values, DLSCT diagnosed the lymph node as metastatic. Axillary ultrasonography (**h**) described the lymph node as suspicious; however, fine-needle aspiration was negative. Sentinel lymph node biopsy contained malignant tumour cells from invasive ductal carcinoma

Conventional and spectral CT datasets were transferred to a dedicated workstation (IntelliSpace Portal 9.0, Philips Healthcare, Best, The Netherlands) for analysis. On conventional CT, the most suspicious ipsilateral lymph node was identified based on size and loss of the fatty hilum and semiautomatically segmented using tumour tracking software by two radiologists with 5 (Thomas Winther Buus) and 2 (Mads Sandahl) years of experience in oncological CT, in consensus. The two radiologists were blinded to all clinical information and did not have access to histopathological diagnosis of the patients’ axillary lymph nodes nor to additional imaging data. In patients without suspicious lymph nodes, one benign-appearing lymph node with a short axis > 0.5 mm was segmented. In addition, one contralateral axillary lymph node (arterial phase) and one inguinal lymph node (venous phase) were segmented as non-metastatic references. From the spectral CT dataset, full series of the chest, abdomen, and pelvis were reconstructed for the following spectral CT parameters: iodine density, mono-energy 40 keV, mono-energy 100 keV, *Z* effective, and virtual non-contrast (VNC). The reconstructed series as well as the lymph node segmentations were transferred to MatLab version 8.6 (MathWorks, Natick, Massachusetts) where the segmentations were applied to the spectral series. From the lymph node segmentations, medians of iodine density, mono-energy 40 keV, mono-energy 100 keV, *Z* effective, VNC, and conventional CT HU from ipsilateral, contralateral, and inguinal lymph nodes were calculated (Fig. 1). A Δ contrast enhancement dataset was calculated as conventional CT (HU) minus VNC (HU). Spectral slope was calculated as (mono-energy 40 keV (HU) minus mono-energy 100 keV (HU))/60 keV.

### Statistical analysis

All statistical analyses were done using STATA Statistics/Data analysis Special Edition version 16.1 (StataCorp, College Station, TX). All values are presented as mean ± standard deviation unless otherwise specified. Normality of the data were assessed visually using normal probability plots which graphically shows departures from normality (*e.g*., outliers, skewness, and kurtosis). Iodine density values and Δ contrast enhancement values were log-transformed, after which they followed a normal distribution. For iodine density, voxels with “0” value were removed from the lymph node histograms, as they were likely derived from adjacent fat. Two-sample *t*-tests were used to compare means. Significant differences were Bonferroni post hoc corrected to adjust for multiple testing. A receiver operating characteristic (ROC) analysis was performed for all spectral CT parameters, and the area under the curve (AUC) was used to compare diagnostic performance. The Youden index [29] was calculated to determine the optimal cutoff values. Sensitivity, specificity, positive predictive value (PPV), and negative predictive value (NPV) based on the optimal cutoff values were calculated using the histopathology report as reference standard. If any lymph nodes were diagnosed as metastatic by histopathology (including micro- and macro- metastases), the most suspicious lymph node segmented on DLSCT was considered metastatic. Sensitivities and specificities of the spectral CT parameters and axillary US were compared using McNemar’s test [30]. A *p* value < 0.05 was considered statistically significant.

## Results

### Patients

**Fig. 2:**
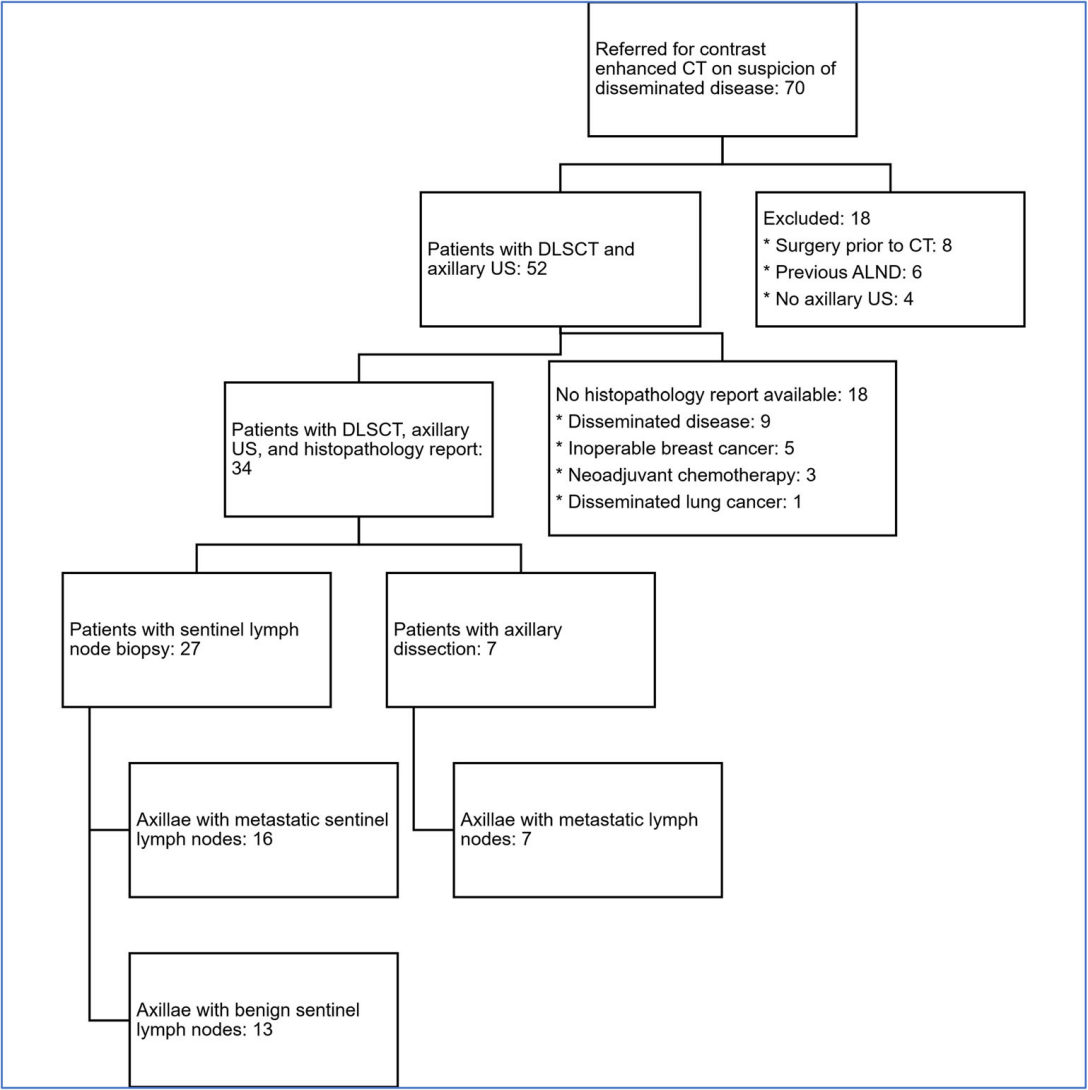
Flowchart showing the patient enrolment. A total of 29 sentinel lymph node biopsies were performed in 27 patients since two patients had bilateral breast cancer. *ALND* Axillary lymph node dissection, *DLSCT* Dual-layer spectral CT, *US* Ultrasonography

From April 2018 to November 2019, 70 women with primary or recurrent breast cancer were consecutively enrolled (Fig. 2). Eighteen patients were excluded: eight had surgery prior to DLSCT, six had previously undergone ALND, and four did not undergo axillary US. Of the remaining 52 patients, 18 did not undergo surgery and thus had no histopathology report available, thus, leaving 34 patients with a DLSCT examination, axillary US examination, and available histopathology report. Two patients had bilateral breast cancer which resulted in 36 axillae being assessed. In the 36 axillae, 29 SNB and 7 ALND were performed within 4 weeks from the DLSCT examination. Twenty-three of 36 axillae had lymph node metastases based on the histopathology report. One axilla had micro-metastases only that were considered metastatic. Patient demographics are shown in Table 1.

**Table 1:** Patient demographics and clinical data

Patient demographics	
Number of patients	34
Number of axillae	36
Age (years mean ± standard deviation)	60.3 ± 15.7
Side	
Right	18
Left	14
Bilateral	2
Patient history	
Primary breast cancer	21
Recurrent ipsilateral breast cancer	9
Previous contralateral breast cancer	4
Type of breast cancer	
Invasive ductal carcinoma	29
Invasive lobular carcinoma	2
Combined	2
Apocrine carcinoma	1
Oestrogen receptor-positive tumour	
Yes	26
No	8
HER2-positive tumour	
Yes	7
No	27
Previous ipsilateral radiotherapy	
Yes	6
No	28
Previous adjuvant chemotherapy	
Yes	7
No	27
Sentinel lymph node biopsy	29
Metastatic	16
Non-metastatic	13
Axillary lymph node dissection	7
Metastatic	7
Non-metastatic	0

*HER2* Human epidermal growth factor receptor 2

#### Quantitative analysis

**Fig. 3:**
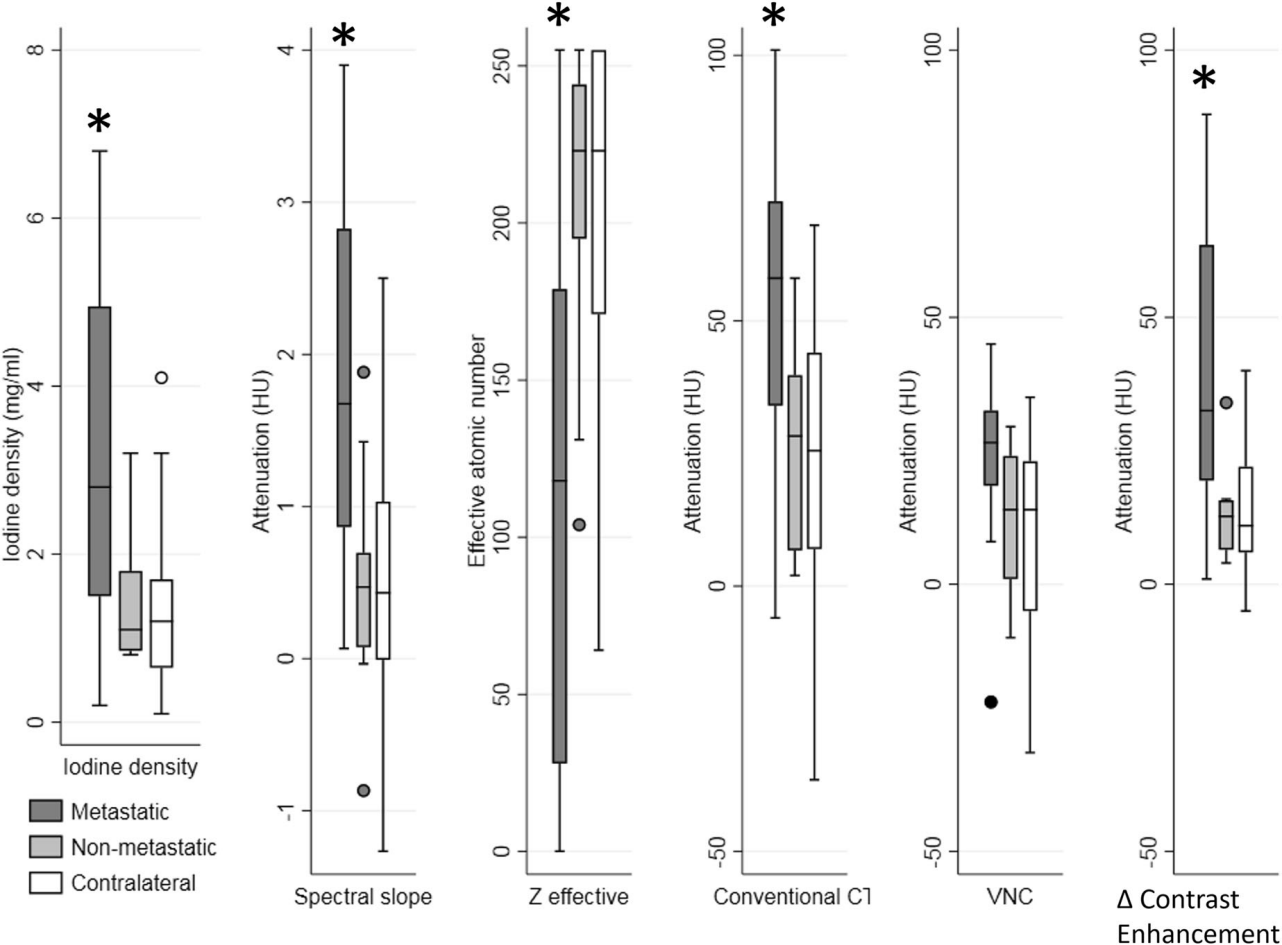
Box chart of quantitative dual-layer spectral CT parameters from metastatic, non-metastatic and contralateral axillary lymph nodes. Metastatic lymph nodes had significantly different iodine density, spectral slope, *Z* effective, Δ contrast enhancement, and conventional CT HU compared to both non-metastatic ipsilateral and contralateral lymph nodes as denoted by the asterisk. *CT* Computed tomography, *VNC* Virtual non-contrast

Box plots of metastatic, non-metastatic ipsilateral, and contralateral lymph nodes for all spectral CT parameters are shown Fig. 3, Median iodine density, spectral slope, *Z* effective, conventional CT HU, and Δ contrast enhancement were significantly different between metastatic and non-metastatic lymph nodes (Table 2). VNC showed no significant difference between metastatic and non-metastatic lymph nodes. There were no significant differences between non-metastatic ipsilateral and non-metastatic contralateral lymph nodes for any parameter. Portal-venous phase inguinal lymph nodes were significantly different from arterial phase contralateral axillary lymph nodes for all parameters except VNC (Table 3).

**Table 2:** Quantitative dual-layer spectral CT parameters from metastatic and non-metastatic ipsilateral axillary lymph nodes

	Median	*p* value
Iodine density (mg/mL)		0.021
Metastatic ipsilateral	2.47 ± 0.23	
Non-metastatic ipsilateral	1.25 ± 0.16	
Spectral slope		< 0.001
Metastatic ipsilateral	1.83 ± 1.18	
Non-metastatic ipsilateral	0.48 ± 0.70	
*Z* effective		< 0.001
Metastatic ipsilateral	111 ± 84.3	
Non-metastatic ipsilateral	209 ± 48.1	
Conventional CT HU		< 0.01
Metastatic ipsilateral	53.9 ± 29.3	
Non-metastatic ipsilateral	26.1 ± 19.1	
VNC (HU)		0.063
Metastatic ipsilateral	24.5 ± 14.3	
Non-metastatic ipsilateral	11.8 ± 13.3	
Δ contrast enhancement (HU)		< 0.01
Metastatic ipsilateral	29.2 ± 2.62	
Non-metastatic ipsilateral	10.8 ± 1.81	

*CT* Computed tomography, *VNC* Virtual non-contrast

**Table 3:** Quantitative dual-layer spectral CT parameters from non-metastatic lymph nodes in arterial phase (contralateral axilla) and portal-venous phase (inguinal lymph nodes)

	Median	*p* value
Iodine density (mg/mL)		< 0.001
Non-metastatic contralateral	0.93 ± 0.27	
Inguinal lymph node	3.77 ± 0.14	
Spectral slope		< 0.001
Non-metastatic contralateral	0.51 ± 0.83	
Inguinal lymph node	2.36 ± 0.91	
*Z* effective		< 0.001
Non-metastatic contralateral	205 ± 53.8	
Inguinal lymph node	79 ± 42.7	
Conventional CT HU		< 0.001
Non-metastatic contralateral	24.4 ± 26.4	
Inguinal lymph node	69.7 ± 26.6	
VNC (HU)		
Non-metastatic contralateral	9.4 ± 19.2	0.092
Inguinal lymph node	16.8 ± 14.8	
Δ contrast enhancement (HU)		< 0.001
Non-metastatic contralateral	12.6 ± 2.2	
Inguinal lymph node	50.6 ± 1.4	

Data are given as mean ± standard deviation

*CT* Computed tomography, *VNC* Virtual non-contrast

### ROC analysis

**Fig. 4:**
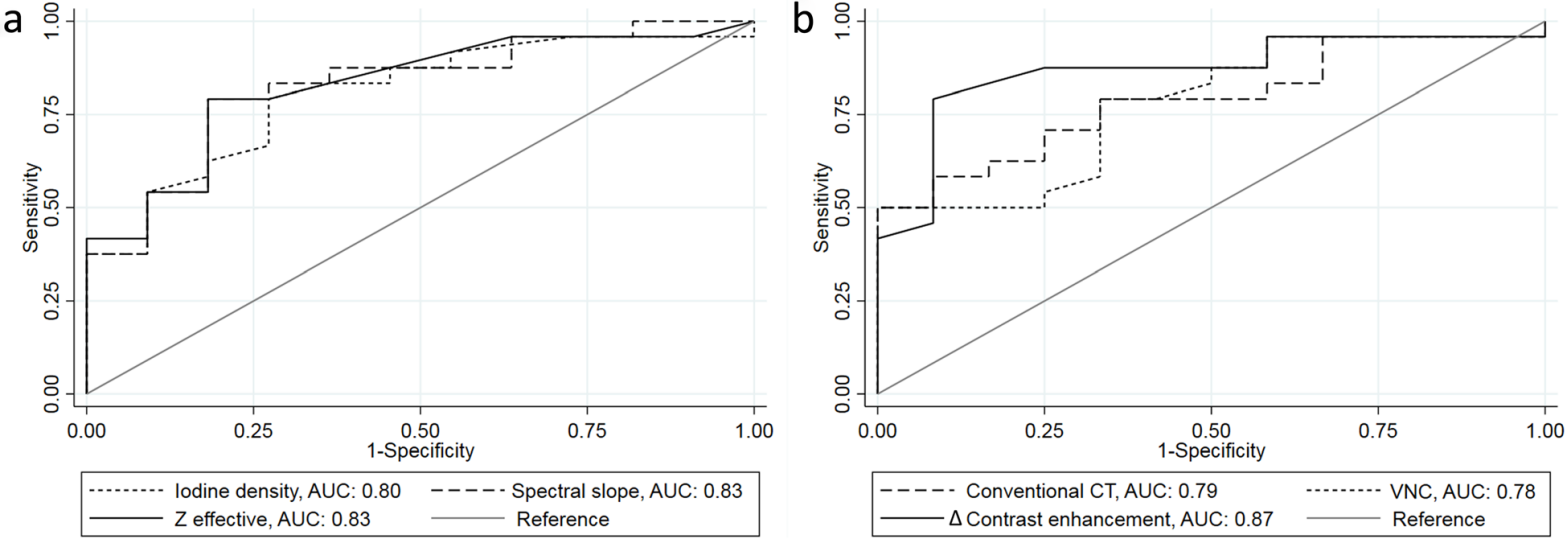
Receiver operating characteristics curves of quantitative dual-layer spectral computed tomography parameters for differentiating metastatic and non-metastatic axillary lymph nodes. *AUC* Area under the curve, *VNC* Virtual non-contrast

ROC curves for all spectral CT parameters are shown in Fig. 4. Table 4 shows cutoff values and AUCs based on the ROC analysis. There were no significant differences in AUC between any spectral CT parameter.

**Table 4:** Diagnostic performance for axillary ultrasonography and quantitative dual-layer spectral CT parameters for differentiating metastatic and non-metastatic axillary lymph nodes

	Cutoff	AUC	Sensitivity	Specificity	PPV	NPV
Axillary ultrasound		0.88	0.75	1.00	1.00	0.67
Iodine density (mg/mL)	1.5	0.80	0.79	0.73	0.83	0.62
Spectral slope	0.82	0.83	0.79	0.83	0.90	0.63
*Z* effective	183	0.83	0.79	0.83	0.90	0.67
Conventional CT HU	53	0.79	0.58	0.92	0.93	0.52
VNC (HU)	18	0.78	0.79	0.67	0.83	0.62
Δ contrast enhancement (HU)	17	0.87	0.79	0.92	0.95	0.69

*CT* Computed tomography, *VNC* Virtual non-contrast. *AUC* Area under the curve, *NPV* Negative predictive value, *PPV* Positive predictive value

Using the cutoff values derived from the ROC analysis yielded the sensitivity, specificity, PPV, and NPV as shown in Table 4. The highest sensitivity, specificity, and AUC were observed for Δ contrast enhancement. Δ contrast enhancement significantly increased sensitivity compared to conventional CT HU (*p* = 0.027). There were no differences in sensitivity between any other parameter. Iodine density, spectral slope, *Z* effective, and Δ contrast enhancement showed no significant differences for AUC, sensitivity, or specificity between them.

**Fig. 5:**
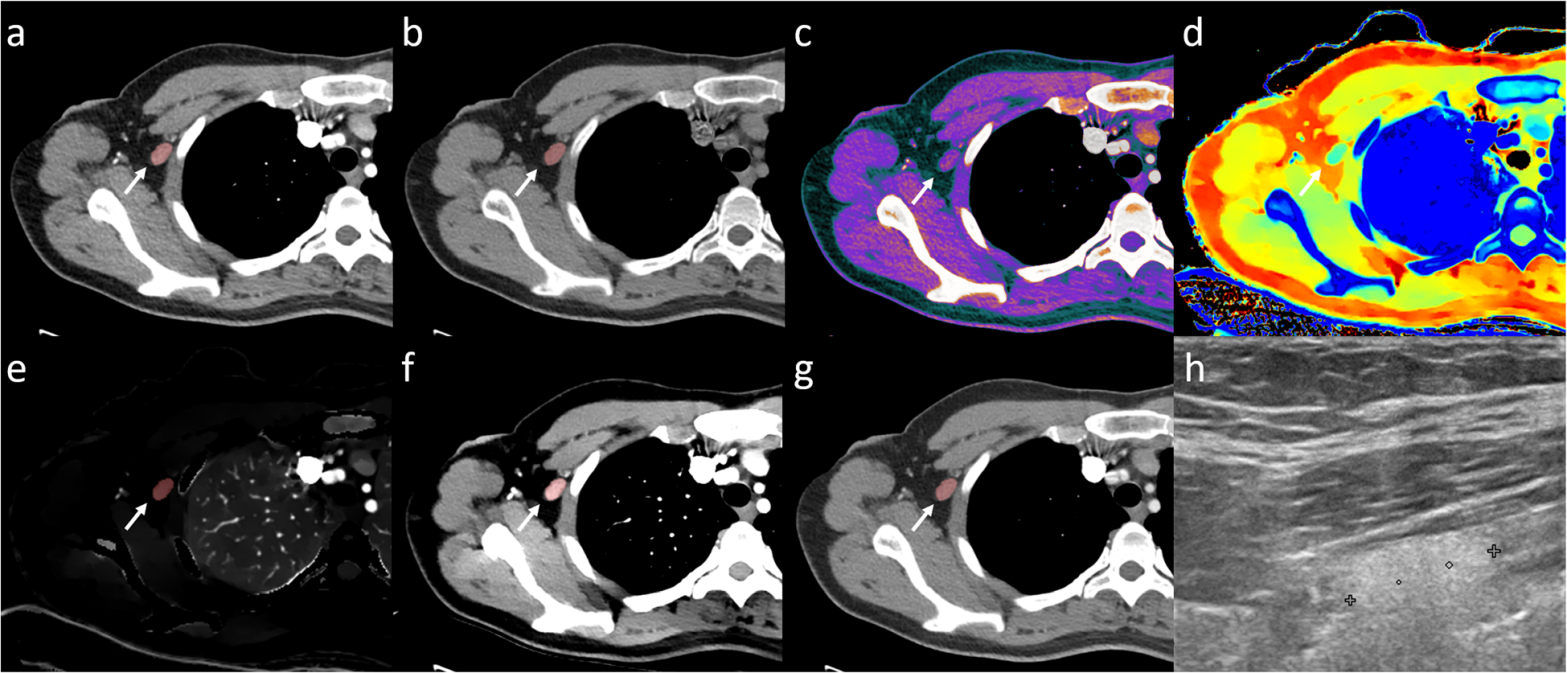
Arterial phase dual-layer spectral computed tomography (DLSCT) examination of a woman with a high attenuating lymph node in the right axilla. The lymph node shows high conventional CT HU (**a**), Δ contrast enhancement (**c**) (conventional CT (HU) (**a**) minus virtual non-contrast (HU) (**b**)), low Z effective (**d**), high iodine density (**e**), and spectral slope ((mono-energy 40 keV (**f**) − mono-energy 100 keV (**g**))/60 keV). Based on the cutoff values, DLSCT diagnosed the lymph node as metastatic. Axillary ultrasonography (**h**) described the lymph node as non-suspicious and consistent with silicone uptake from a previously known breast implant leakage. Sentinel lymph node biopsy was negative for metastatic disease but showed significant silicone content

**Fig. 6:**
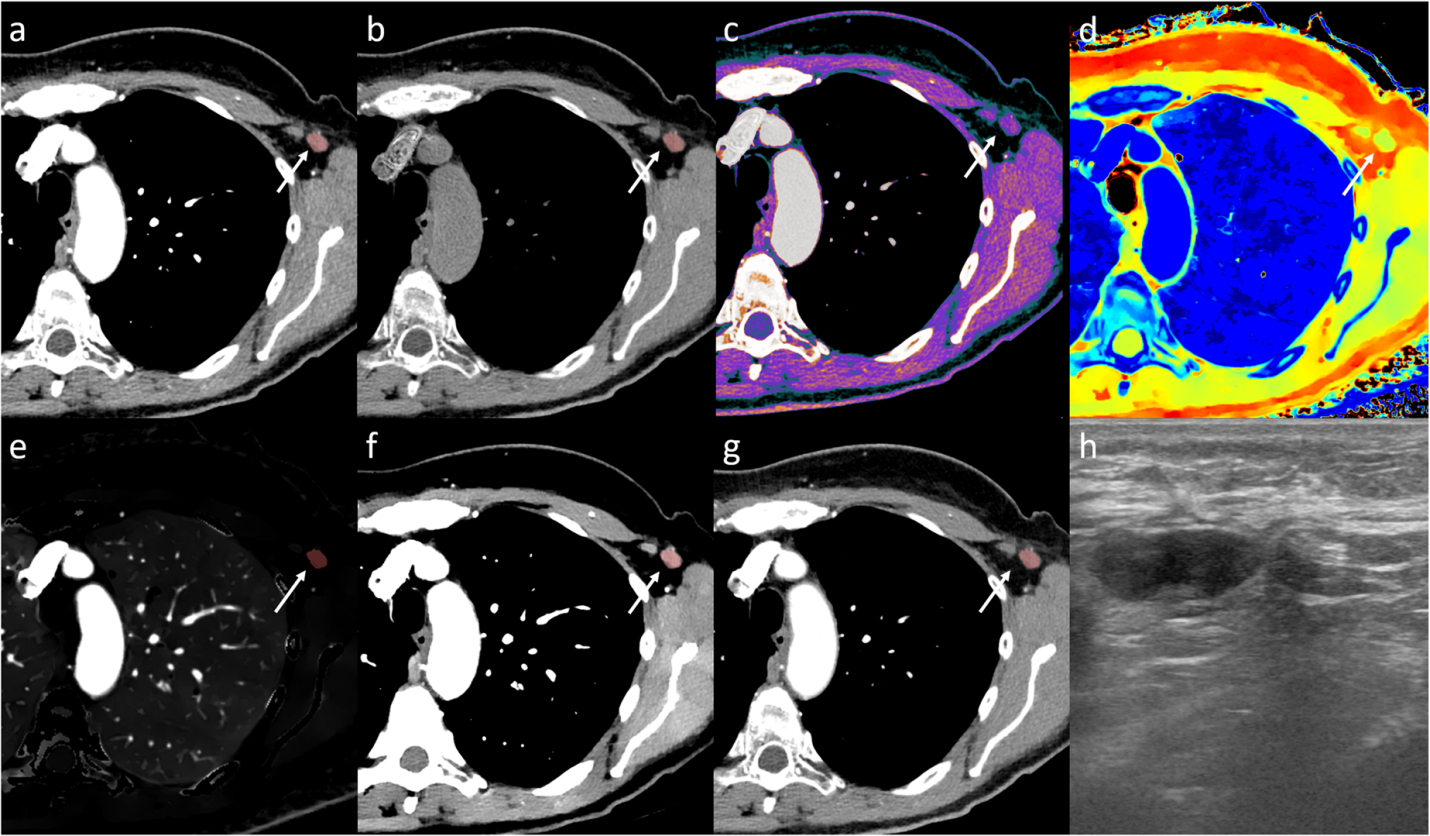
Arterial phase dual-layer spectral CT (DLSCT) examination of a woman with left-sided breast cancer and a lymph node in the left axilla. The lymph node does not show increased conventional CT HU (**a**), Δ contrast enhancement (**c**) (conventional CT (HU) (**a**) minus virtual non-contrast (HU) (**b**)), iodine density (**e**), or spectral slope ((mono-energy 40 keV (**f**) minus mono-energy 100 keV (**g**))/60 keV). However, *Z* effective (**d**) is decreased. Based on the cut-off values, both Δ contrast enhancement and conventional CT HU diagnosed the lymph node as non-metastatic. Axillary ultrasonography (**h**) described the lymph node as suspicious, and fine-needle aspiration contained malignant tumour cells. Sentinel lymph node biopsy contained malignant tumour cells from invasive ductal carcinoma

Axillary US had significantly higher specificity compared to iodine density (*p* = 0.049) and VNC (*p* = 0.045) without significant difference in sensitivity. There were no significant differences between Δ contrast enhancement and axillary US for sensitivity (*p* = 1.000) or specificity (*p* = 0.320). Δ contrast enhancement had one false positive (Fig. 5) whilst axillary US had zero. A total of five false negatives were seen for Δ contrast enhancement (Fig. 6) compared to six false negatives for axillary US.

## Discussion

In the present study, we used quantitative spectral CT parameters derived from DLSCT to diagnose axillary lymph node metastases in breast cancer patients. DLSCT significantly improved sensitivity compared to conventional CT HU and had similar sensitivity and specificity such as axillary US with FNA. Metastatic lymph nodes showed significantly higher iodine density, spectral slope, and Δ contrast enhancement whilst *Z* effective was lower compared to non-metastatic lymph nodes.

The best diagnostic performance was found for Δ contrast enhancement using a cutoff value of 17 HU, which resulted in a sensitivity, specificity, and AUC of 0.79, 0.92, and 0.87, respectively. In a previous study, Tawfik et al. [18] found a sensitivity of 0.90, specificity of 0.78, and an AUC of 0.90 for Δ contrast enhancement to detect cervical lymph node metastases from squamous cell carcinoma using dual-source DECT. The differences, albeit minor, in sensitivity and specificity between our study and the study by Tawfik et al. could be due to them scanning the lymph nodes in the venous phase. Since Δ contrast enhancement was the difference in HU between contrast-enhanced images and VNC images, it could potentially also be obtained by scanning the patients before and after contrast administration.

Compared to the histopathology report, Δ contrast enhancement missed five metastatic lymph nodes whilst axillary US missed six, three of them being from the same patient. The three lymph node metastases that were detected by Δ contrast enhancement but not by axillary US all had a short axis < 10 mm with morphologically benign appearance. Δ contrast enhancement only had one false positive in a high attenuating lymph node (Fig. 5). The histopathology report showed silicone content from a leaked breast implant and the lymph node was correctly diagnosed as non-metastatic by axillary US. However, in contrast to DLSCT, the breast radiologists performing the axillary US had access to the patient records and were aware of the leaking breast implant. DECT is able to visualise silicone and has been shown to be able to detect leaks from ruptured breast implants [31, 32]. Since the patient’s records would be available in a clinical setting, it is likely that a clinical reading of the DLSCT would correctly diagnose the silicone uptake in the lymph node. The high specificity and PPV found in our study (0.92 and 0.95, respectively) are particularly promising since, if the high specificity and PPV could be replicated in a larger patient cohort, > 3 positive nodes on DLSCT could potentially mean that SNB could be avoided and the patient could proceed directly to surgery and ALND. Because DLSCT, in contrast to axillary US, exposes the patient to ionising radiation, it is unlikely to become a standard part of the diagnostic workup in all women with loco-regional breast cancer. However, quantitative DLSCT could potentially have a place in the diagnostic workup of women with a high a priori risk of metastatic disease where contrast-enhanced CT is already indicated (*e.g*., patients with palpable lymph nodes).

Whilst iodine density had lower diagnostic performance than Δ contrast enhancement, it still showed sensitivity comparable to axillary US. The optimal cutoff for distinguishing metastatic and non-metastatic lymph nodes were calculated to 1.5 mg/mL. In a recent study by Volterrani et al. [33], they found a cutoff value of 1.7 mg/mL iodine in the late arterial phase for differentiating malignant and benign breast lesions using a rapid tube potential switching DECT protocol. Based on these results, it seems that adopting a threshold of 1.5–2.0 mg/mL iodine could be useful both for assessing breast lesions as well as lymph nodes.

Significant differences in iodine density, spectral slope, *Z* effective, conventional CT HU, and Δ contrast enhancement were observed between non-metastatic contralateral lymph nodes in arterial phase and non-metastatic inguinal lymph nodes in portal-venous phase. This was expected as the spectral CT parameters all depend on the iodine uptake in the lymph nodes which is phase dependent. These findings are in concordance with previous studies which have shown that iodine density, spectral slope, and *Z* effective are lower in arterial phase compared with venous phase for both metastatic and non-metastatic lymph nodes [19, 23]. In a study by Zhang et al. [23] where DECT was performed both in arterial and venous phase, spectral parameters from axillary lymph nodes in the venous phase showed higher diagnostic performance than in the arterial phase.

Some previous studies have tried to compensate for phase and differences in the patients’ circulation by normalising the iodine density or *Z* effective to the internal carotid artery [19] or aorta [20, 23]. We did not normalise our data to the arterial attenuation, since our aim was to assess the diagnostic performance of the available images in a clinical setting. Instead, all our axillary lymph node parameters were obtained in the arterial phase 15 s after a threshold of 150 HU was measured in the descending aorta to minimise differences in circulation. The one spectral CT parameter that does not depend on iodine uptake—the VNC—was not significantly different between phases. This shows that the phase has to be considered when quantifying spectral CT parameters and is a challenge for adopting diagnostic thresholds since DECT protocols vary amongst institutions.

The strengths of our study include the prospective patient enrolment and the use of histopathological examination of the lymph node as reference standard. The lymph nodes were semi-automatically segmented using tumour tracking software by two radiologists in consensus to reduce the subjective nature of manual drawing of regions of interest. We used a DLSCT scanner that acquires spectral CT datasets without the need for changing the scan protocol, where previous studies assessing quantitative spectral CT parameters for detecting lymph node metastases have used dual-source CT or single-source CT with rapid tube potential switching. The reconstruction of the spectral base images took less than five minutes after which all spectral CT datasets were available making it feasible to implement in the clinic.

Our study has some limitations. First, we did not correlate lymph nodes from DLSCT with the histopathology report on a node-to-node basis but used a region-to-region correlation instead. Second, all axillary lymph nodes were scanned in arterial phase. It would have been interesting to compare the diagnostic performance between contrast phases using DLSCT. Finally, our study population was small, and only 13/36 axillae had benign histopathology. Since the patients were referred for contrast-enhanced CT of the chest, abdomen, and pelvis on a suspicion of disseminated disease, the a priori risk of having axillary lymph node metastases was increased in this patient cohort compared to the general patient population. In order to generalise the results, multi-centre studies with larger patient cohorts are needed.

In conclusion, DLSCT improved the sensitivity to detect lymph node metastases compared to conventional CT HU with similar sensitivity and specificity as US with FNA and had a very high PPV. DLSCT is a promising quantitative technique for identifying lymph node metastases and could potentially reduce the need for SNB in patients with positive findings. Future studies should aim to validate the diagnostic performance of DLSCT on a node-to-node basis in a larger cohort.

## Data Availability

The datasets used and/or analysed during the current study are available from the corresponding author on reasonable request.

## Abbreviations

ALND: Axillary lymph node dissection
AUC: Area under the curve
DECT: Dual-energy computed tomography
DLSCT: Dual-layer spectral computed tomography
FNA: Fine needle aspiration
NPV: Negative predictive value
PPV: Positive predictive value
ROC: Receiver operating characteristic
SNB: Sentinel lymph node biopsy
US: Ultrasonography
VNC: Virtual non-contrast
